# Basal Level of Autophagy Is Increased in Aging Human Skin Fibroblasts *In Vitro*, but Not in Old Skin

**DOI:** 10.1371/journal.pone.0126546

**Published:** 2015-05-07

**Authors:** Dino Demirovic, Carine Nizard, Suresh I. S. Rattan

**Affiliations:** 1 Laboratory of Cellular Ageing, Department of Molecular Biology and Genetics, Aarhus University, Aarhus, Denmark; 2 LVMH Research, St. Jean de Braye, France; National Centre for Scientific Research, 'Demokritos', GREECE

## Abstract

Intracellular autophagy (AP) is a stress response that is enhanced under conditions of limitation of amino acids, growth factors and other nutrients, and also when macromolecules become damaged, aggregated and fibrillated. Aging is generally accompanied by an increase in intracellular stress due to all the above factors. Therefore, we have compared the basal levels of AP in serially passaged human facial skin fibroblasts undergoing aging and replicative senescence *in vitro*, and *ex vivo* in the skin biopsies from the photo-protected and photo-exposed area of the arms of 20 healthy persons of young and old ages. Immunofluorescence microscopy, employing antibodies against a specific intracellular microtubule-associated protein-1 light chain-3 (LC3) as a well established marker of AP, showed a 5-fold increase in the basal level of LC3 in near senescent human skin fibroblasts. However, no such age-related increase in LC3 fluorescence and AP could be detected in full thickness skin sections from the biopsies obtained from 10 healthy young (age 25 to 30 yr) and 10 old (age 60 to 65 yr) donors. Furthermore, there was no difference in the basal level of LC3 in the skin sections from photo-protected and photo-exposed areas of the arm. Thus, in normal conditions, the aging phenotype of the skin cells in culture and in the body appears to be different in the case of AP.

## Introduction

Optimal stress response (SR) is an essential aspect of the biological property of homeostasis/ homeodynamics [1,2]. Among the major intracellular SR pathways, autophagy (AP) is a response to insufficiency of nutrients; and is being increasingly recognized for its role in survival, aging, longevity and age-related pathology, including cancer [3,4]. AP is a regulatory “self-eating” process, which involves cytoplasmic degradation of macromolecules and other large compartments of the cell, such as the mitochondria. Under normal conditions, AP operates constitutively at a basal level concurrent with lysosomes and proteasomes [5]. However, AP is enhanced under conditions of limitation or deprivation of amino acids, growth factors and other nutrients, or when macromolecules become damaged, aggregated, fibrillated or in some other way modified and are not used by the cells. Thus, AP is one of the survival mechanisms for cells during extrinsic and intrinsic stress, whose biological consequences depend on the level of the stress. For example, whereas too much AP can lead to cell death, moderately enhanced AP during dietary restriction slows down aging and increases the lifespan of cells and organisms [6]. Since the process of aging is characterized by a progressive occurrence and accumulation of damage, including protein aggregation, fibrillation, mitochondrial damage and reduced efficiency of energy production [7,8], it is relevant to find out if the basal level of AP is altered during aging.

One of the widely used approaches to study AP is the appearance and disappearance of a specific intracellular microtubule-associated protein 1 light chain 3 (LC3), which can be used as a marker for the autophagic flux [9]. LC3 is a cytoplasmic and constitutively expressed protein, which is cleaved at its C-terminus by Atg4 protease generating the cytosolic form LC3-I. It is then conjugated to phosphatidylethanolamine (PE) in a ubiquitin-like reaction and the lipidated form of LC3, known as LC3-II, is attached to the autophagosome membrane. The formation of a cytoplasmic double membrane, the so-called phagophore, probably originating from the endoplasmatic reticulum, then expands and forms a closed sphere called the mature autophagosome, which is recognized by the lysosomal associated membrane proteins (LAMPs), and the contents of the autophagosome, including LC3-II, are degraded by the lysosomal enzymes [10].

We have studied and compared the basal levels of LC3-II as an indicator of autophagic flux in serially passaged human facial skin fibroblasts undergoing aging and replicative senescence *in vitro*, and *ex vivo* in the skin biopsies from healthy persons of different ages. The cell culture model system of serial passaging and cellular aging *in vitro*, also known as the Hayflick system, is a widely used system for understanding the phenomenological and mechanistic aspects of aging [11,12]. However, not all aspects of aging are reflected in this model system in an exact manner, and some changes observed *in vitro* may be exaggerated or diminished in the normal situation *in vivo*. Therefore, comparative studies are important to understand and resolve the significance of age-related changes in different model systems and conditions.

## Materials and Methods

Facial adult skin fibroblast cell strain designated as FSF-1, was established from a healthy 40-yr old woman´s eye-lid, at LVMH-Research, St. Jean de Braye, France, and stored frozen in liquid nitrogen at passage 2, as described previously [13]. FSF-1 cultures were grown and maintained in plastic tissue culture flasks (NUNC, Roskilde, Denmark), in an incubator at 37°C, 95% relative humidity and 5% CO_2_, using Dulbecco´s Modified Eagle’s Medium, (DMEM; Biowhittaker, Viviers, Belgium), supplemented with 10% bovine Fetal Calf Serum (FCS; Biological Industries, Beit, Haemek, Israel), and 100 U/mL penicillin/streptomycin (Biowhittaker, Viviers, Belgium). For sub-culturing, monolayer cultures near confluency (90–95% of the growth surface covered by cells) were trypsinized with 0.25% trypsin-EDTA, and distributed into new cell culture flasks. Cells were serially sub-cultured or passaged at 1:2 or 1:4 split ratio until they stopped dividing, became irreversibly growth arrested and entered a state of senescence. In order to estimate the proliferative lifespan of FSF-1 cells, 1 or 2 passages (P) were added to the age of the cultures at each sub-culturing at 1:2 or 1:4 split ratio, respectively.

Human skin tissues were collected from 10 female donors each in two different age groups (25–30 years old and 60–65 years old). Punch biopsies were taken from both the upper and lower arm, representing the photo-protected and photo-exposed areas of the skin. These 40 samples were taken from Asian and Caucasian donors in the month of February in Hamburg, Germany, thus avoiding having recently sun-exposed or sun-burnt skin. None of the donors had any medical condition or used any medications. The biopsies were flash-frozen in liquid nitrogen immediately after excision.

With respect to the ethical permissions, for studies using skin biopises, the principle requirements of the Declaration of Helsinki were taken into account to protect the rights, safety and well-being of subjects participating in the study. Before initiating the studies, the investigator (CN) had obtained written consent from the participants, and full approval from the Freiburg Ethics Commission International for the protocol, protocol amendment(s), if applicable, and the subject informed consent form. The Freiburg Ethics Commission International is the Regional Ethics Committee and is responsible for approving ethical aspects of our research at our institution (LVMH). All participants who provided their skin biopsies in this research provided their written informed consent to participate in this study and for their data to be used for research purposes. For cell culture studies, skin samples were collected from adult patients, undergoing plastic surgery performed by independent plastic surgeons and were considered as ‘‘waste”, and thus were exempt from any further approval. Even in this case, verbal informed consent from the participants that provided their waste tissues for research purposes was obtained. All the waste tissues were completely de-identified, including removal of any and all demographic, genetic and health information prior to being handed over to the researchers. All the data in this study was anonymized *(all relevant documents available at LVMH-Research*, *St*. *Jean de Braye*, *France)*.

### Cell Lysis and Western Blotting

FSF-1 cells at different passage levels were harvested by scrapping with a rubber scrapper in ice-cold PBS, centrifuged for 5 min at 300 x *g*, and the cell pellets were dissolved in lysis buffer (20 mM Tris-HCl with 150 mM NaCl pH 7.6, 2% Triton X-100, 2mM phenylmethanesylfonyl in isopropanol, 1x complete Protease Inhibitor Cocktail (Roche, Basel, Switzerland), 2.5 mM MgCl_2_, 0.5 mM CaCl_2_ and 10 μg/mL DNase). Protein concentrations were determined by the Bradford method, and 20 μg of total protein from each sample was separated by polyacrylamide-gel-electrophoresis (Precasted 8–12% gels from BIORAD). The separated proteins were subsequently transferred to a nitrocellulose membrane before they were immunolabeled with specific antibodies. For detection of autophagy, polyclonal antibodies for the marker LC3 (Catalog number: PM036, MBL Intl.) were used after 1:800 dilution in 1% BSA. The membranes were then processed for chemiluminiscence before they were developed on an X-ray film.

### Immunofluorescence Microscopy

FSF-1 cells were seeded on 4-chamber glass slides (NUNC, Roskilde, Denmark) at a density of 10,000 cells/cm^2^, allowed to attach and stabilize for 24 hr before being fixed with 2% paraformaldehyde, pH 7.4, at room temperature (RT; about 21°C) for 5 min and permeabilized with 100 μg/mL Digitonin. For studies on skin tissues, the tissue samples were cut frozen, using a microtome, at a thickness of 8–10 μm and left to dry at 37°C for 20 min. The tissue samples were then washed three times in PBS.

Both the cells and the skin sections were then blocked in 1% BSA in PBS at RT for 30 min before they were incubated with primary antibodies, for at least 1 hr. The antibody used was the same as for LC3 detection described above for Western blotting, however the dilution for tissue and fixed cells was 1:200 in 1% BSA. The samples were then washed 3 times before they were incubated with the appropriate secondary antibodies. The samples were subsequently analyzed by a confocal fluorescence microscope.

### Data analysis

Microscopic analysis was done using either a Leica DM3000 B inverted fluorescence light microscope or a Leica SP5 confocal laser scanning microscope for fluorescence images. For plain light and phase contrast microscopy a Zeiss Axiovert 25 Inverted microscope was used together with Zeiss Axiovision software. Fluorescence images were afterwards quantified using Leica QWin software, and applied to all images of interest. All microscopic images were taken at the same camera settings, exposure time, and same laser intensity.

Western blot films were scanned on a Cannon Flatbed scanner, and were analyzed using ImageJ (http://rsb.info.nih.gov/ij/) gel analysis software. All the sample bands were quantified in relation to the control band, and furthermore to the equivalent to their respective actin levels. The data analysis was done in Microsoft Excel 2010–13, and the data presentation was completed in Prism GraphPad. All experiments were performed at least three independent times, with cells used at the same passage number. The median of each triplicate set of assays was used as the result in data presentation. We assume that the populations of cells were normally distributed (Gaussian distribution), and since the population sizes were relatively small, standard deviation will be displayed for every possible result.

## Results and Discussion

### Increased basal levels of autophagy during cellular aging *in vitro*

Starting from a frozen ampoule at P2, serially passaged FSF-1 cells, in this series of experiments, achieved a final cumulative P level of 61, considered as 100% lifespan completed in 333 days, as reported previously [13]. Upon serial passaging, FSF-1 exhibited the well-established Hayflick-phenomenon of cellular aging and replicative senescence [14,15], and nearing senescence, FSF-1 cells showed the classical senescent phenotype, such as membrane irregularity, enlarged size, multi nuclei and so on. Furthermore, it was observed that late passage FSF-1 cultures had approximately 90% senescence-specific β-galactosidase (SABG) positive cells, versus less than 1% SABG positive cells in early passage cultures, as expected for this widely used biomarker of aging [16] *(pictures not shown)*.

**Fig 1:**
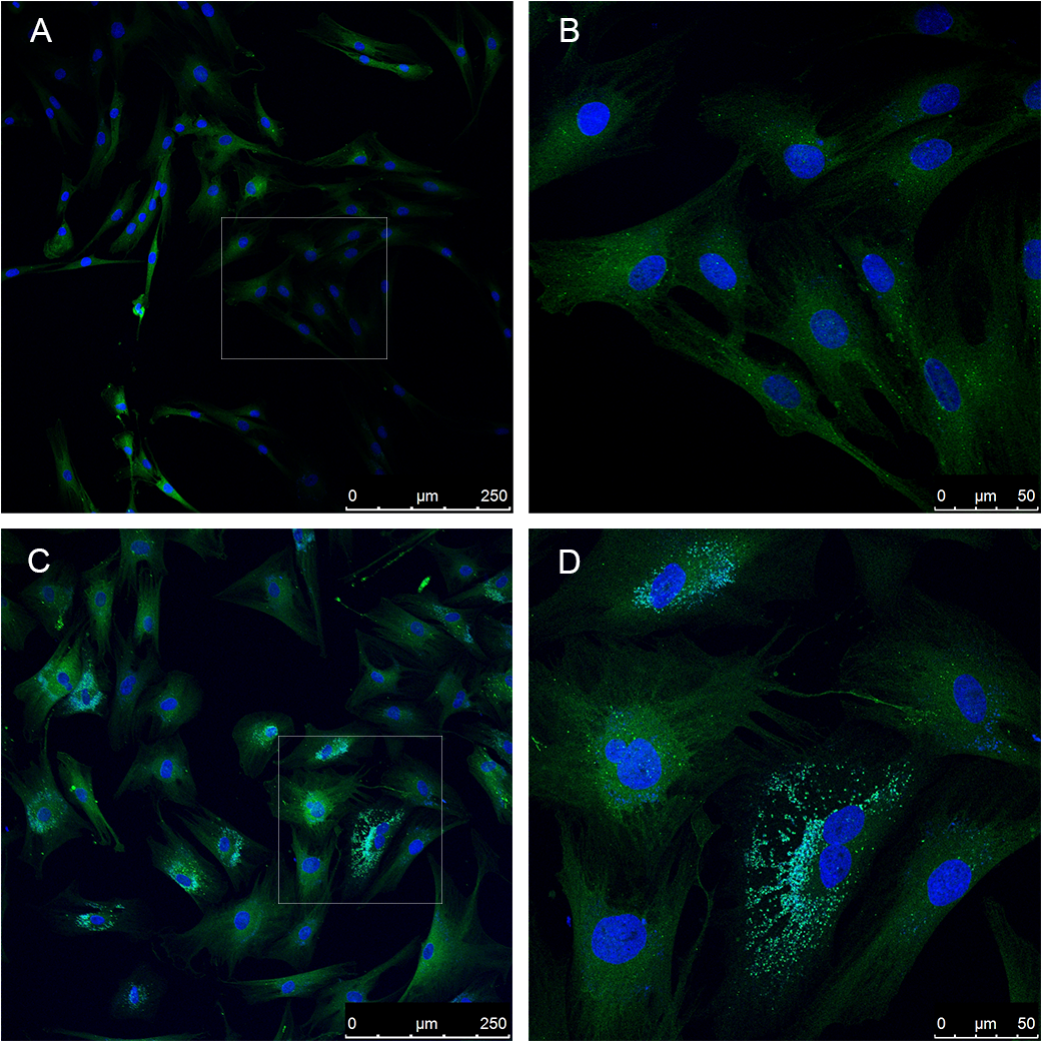
Increased basal levels of autophagy marker LC3 in serially passaged human skin fibroblasts during aging *in vitro*. Fluorescence confocal photomicrographs of FSF-1 cells stained for LC3 (green fluorescence) and DAPI for nuclear detection (blue fluorescence). Scale bars represent the microscopic magnifications as low or high. (A) Early passage (P14) cells showing the general distribution of LC3 in this population. (B) Selected area from early passage FSF-1 cells at high magnification showing few cells and their internal distribution of LC3 in puncta form. (C) Late passage (P55) near senescent FSF-1 cells, and their overall distribution in the population. (D) Selecgted area from late passage near senescent FSF-1 cells at higher magnification showing distinct senescent morphology, enlarged size and multinucleation, and the extranuclear micronuclei.

As for AP, confocal immunofluorescence photomicrographs of early passage young (P14) and late passage (P55; more than 90% lifespan completed) FSF-1 cells stained for AP marker LC3 protein (green) and with DAPI (blue) for nucleic acids are shown in Fig 1. It should be pointed out that in these pictures, it is not possible to distinguish between LC3-I and LC3-II, since the antibodies recognized both types of the protein. Fig 1A and 1B) of early passage cells at low and high magnification, respectively, show low level AP activity, as represented by the presence of LC3 puncta in the cytoplasm. In comparison, late passage senescent cells (Fig 1C and 1D) show much higher basal level of AP activity in terms of LC3 puncta in the cytoplasm. Both early and late passage cells were seeded sparsely and were given fresh culture medium 24 hr prior to staining in order to avoid any other stress, such as crowding and serum-shortage, which may affect the basal levels of AP.

It is obvious from the photomicrographs that any AP activity in early passage cells was mostly limited to larger cells, whereas it was minimal in the small, thin and elongated cells, which is a characteristic of young cells. Since serial passaging-related increase in cell size is a well-established phenotype of cellular aging *in vitro* [12], majority of the cells at late passages are much enlarged and show high AP activity. Furthermore, in senescent cells the cell membrane becomes irregular and some cells exhibit two or more nuclei, which also promote the formation of autophagosomes, and induces autophagy [17]. Quantification of fluorescence photomicrographs for the green fluorescence intensity showed that there was about 5-fold difference between young and old cells.

**Fig 2:**
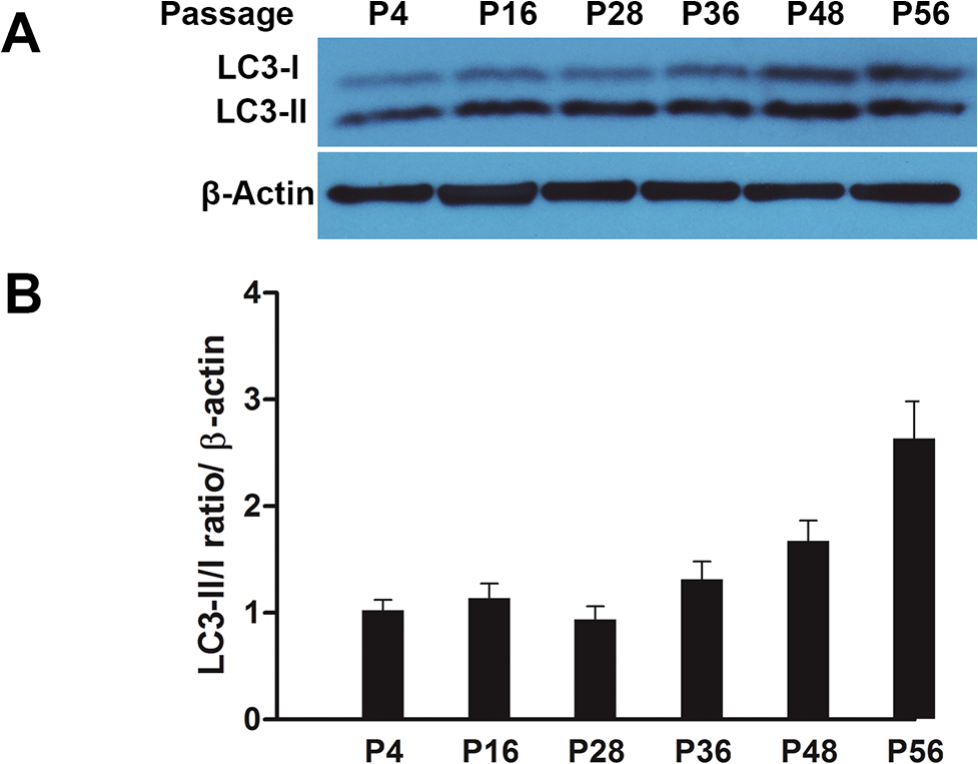
Immunoblotting-based comparison of levels of LC3-I and LC3-II in serially passaged human skin fibroblasts during aging *in vitro*. Cell samples were analyzed at six age points (P4, 16, 24, 36, 44 and 56) covering the whole replicative lifespan. (A) Immunoblot bands for LC3-I and LC3-II, and ß-actin as internal loading control. (B) Quantitative analysis of band intensity using ImageJ gel analysis software; data are presented as LC3-II/LC3-I ratio/ ß-actin.

The basal levels of LC3 were further confirmed by immunoblotting showing a comparison of LC3-I and LC3-II levels at 6 age-points in serially passaged FSF-1 cells (Fig 2). LC3-II/LC3-I ratio at different passage levels relative to β-actin levels of each band confirmed that late passage cells had up to 3-fold higher AP than the early passage cells. It is also noticeable that the increase in the LC3-II/I ratio first appears after P36, which corresponds to more than 50% lifespan completed (Fig 2B). This also indicates that AP is stalled in late passage cells. Such low level AP activity in unstressed and rapidly dividing low passage cells has also been reported for intestinal epithelial cells, adult mesenchymal stem cells and immortalized HEK cells [18–20]. However, these previous studies made no comparison between early and late or low and high passage level cells to show the effects of serial passaging and cellular aging.

Our study is thus perhaps the first one to report that there is a significant increase in the basal level of AP in serially passaged human facial skin fibroblasts. These data further support the general view of age-related increase in intrinsic stress that leads to the compensatory stimulation of various stress responses as an attempt to counteract any harmful effects of stress [1,7]. Some of the main sources of stress during aging of cells *in vitro* are the normal biochemical and metabolic processes, cumulative molecular damage and even cell culturing conditions using plastic culture flasks and high oxygen levels which promote oxidative damage.

### Unchanged basal levels of autophagy in aging skin

**Fig 3:**
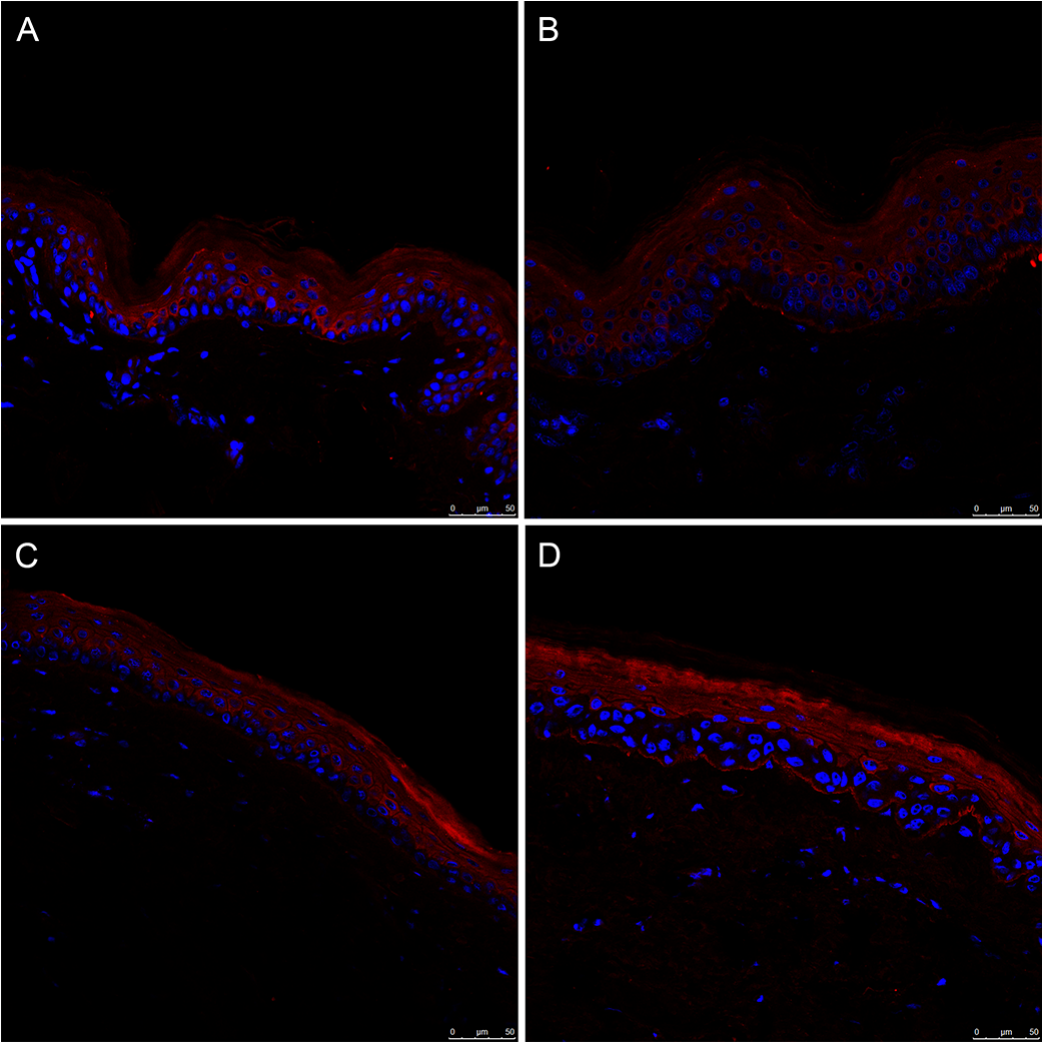
Basal levels of autophagy marker LC3 in full thickness human skin sections from young and old donors. Representative fluorescence confocal photomicrographs for LC3 (red fluorescence) and DAPI is used for nucleic staining (blue fluorescence). (A) skin section from the photo-protected upper arm of a young donor, age 27 yr. (B) skin section from the photo-exposed lower arm of the same young donor. (C) skin section from the photo-protected upper arm of an old donor, age 60 yr. (D): skin section from the photo-exposed lower arm of the same old donor.

Fig 3 shows immunofluorescence photomicrographs of the skin sections from young and old healthy donors, stained for AP marker LC3 protein (red) and with DAPI (blue) for nucleic acids. These 4 pictures are the representative pictures from 40 skin biopsies, from photo-protected (Fig 2A and 2C) and photo-exposed sites (Fig 2B and 2D), obtained from 10 young and 10 old donors, respectively. The pictures show the LC3 as red fluorescence while the nuclei are stained blue with DAPI. There were obvious structural differences in the appearance of the skin from young and old donors in terms of thickness, regularity and other features as described in the scientific literature [21,22].

As regards the basal AP activity in the skin, LC3 levels were relatively higher in the epidermis as compared to the dermis regardless of the donor age (Fig 3).

**Fig 4:**
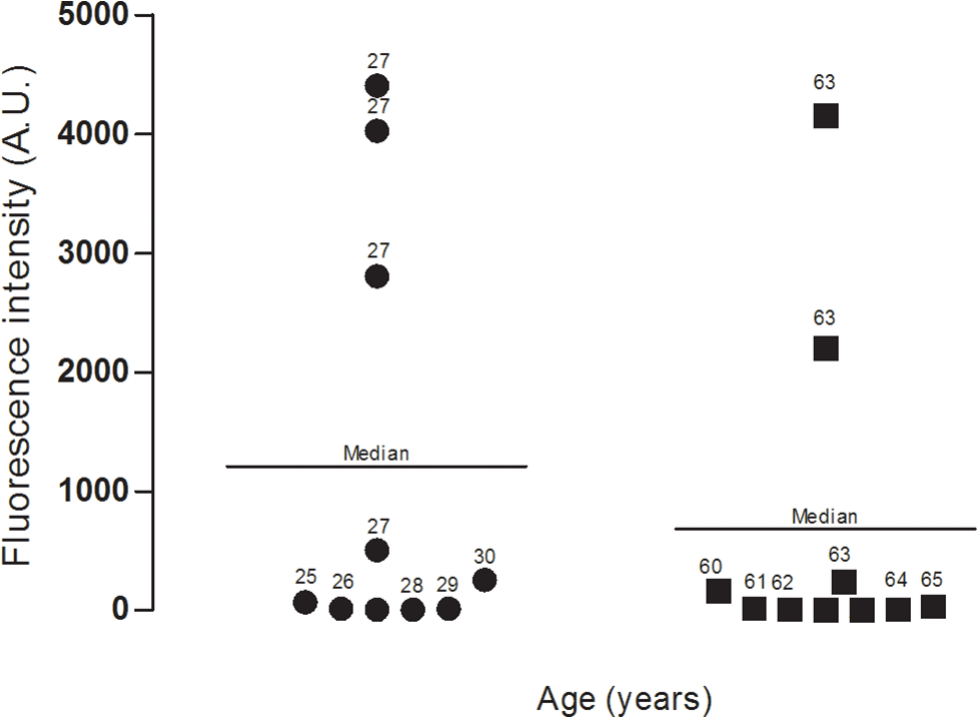
Unaltered basal levels of autophagy marker LC3 in human skin sections from young and old donors. Data represent combined fluorescence levels (arbitrary units) in the skin sections from 10 young and 10 old donors; at least 5 microscopic pictures from each donor were analyzed in this data set. Median value is represented by a line (p = 0.418).

In the epidermis there seems to be an increasing gradient of LC3 fluorescence from the basal layer to the stratum corneum, which mostly consists of dead corneocytes. However, there were no significant differences in the basal level of LC3 in the skin sections either from the young and the old donors or from the photo-protected and photo-exposed areas (Fig 2). Combined quantitative data for the level of LC3 fluorescence, measured in at least 5 photomicrographs from each section from 10 young and 10 old donors, is presented in Fig 4. Although there were some inter-individual variations, there was statistically non-significant difference (p = 0.418) between the young and the old donors in this study. Furthermore, we did not find any correlation between photo-exposed and photo-protected skin regardless of donor age. Even though there might be individuals whose skin sections showed tremendous increase in the levels of LC3, we did not find any correlation between the donor age and the basal level of LC3.

### Conclusions

Equating the results of studies made on skin cells in culture and the tissue sections from the biopsies from the skin may only partly clarify the complexity of a biological process such as aging. Whereas cell culture-based results clearly show that there is an age-related increase in the basal AP activity level in human skin fibroblasts, such an increase is not discernible in the tissue sections obtained from old donors as compared with those from young donors. This apparent discrepancy does not mean that the AP machinery, usually known as AP flux, is not being altered with increasing donor age. For example, although we had analyzed the extent of AP flux by using lysosomal blockers in serially passaged FSF-1 cells at young and near senescent stages, such experimental induction of AP flux was not possible to perform in the frozen section of the human skin. Similarly, in this study, it was not possible to determine the level of LC3 by immunoblotting of the frozen sections of the skin. Therefore, in follow up studies, determination of levels of LC3 protein and mRNA transcripts during aging *in vitro* and *in vivo*, and the extent of AP flux in freshly obtained skin biopsies can provide useful further information.

It is possible that under additional stressful conditions, induction of AP and other stress responses, is reduced both *in vitro* and *in vivo* as a phenotype of impaired survival ability during aging. Further studies are required for addressing that issue. Therefore, for *in vivo* situations, it is important to make use of more dynamic methods applicable to the living tissue or organs. The data presented above do not indicate that the Hayflick system of human skin cell aging *in vitro* is not a relevant and convenient model system to study certain aspects of aging. Our results clearly show that there is an age-related increase in the intrinsic stress levels of cells, as indicated by the increased basal levels of one of the major stress responses, during their limited proliferative lifespan. These results also remind that extrapolating conclusions derived from model systems of aging *in vitro* to the tissue and organ systems *in vivo* is not a simple and straight forward matter, and one should be fully aware and cautious of the boundaries of usefulness of the particular model system, especially while trying to understand a highly complex and multi-variant process, such as aging.
